# Enhanced Late I_Na_ Induces Intracellular Ion Disturbances and Automatic Activity in the Guinea Pig Pulmonary Vein Cardiomyocytes

**DOI:** 10.3390/ijms25168688

**Published:** 2024-08-09

**Authors:** Taro Saito, Mahiru Suzuki, Aiko Ohba, Shogo Hamaguchi, Iyuki Namekata, Hikaru Tanaka

**Affiliations:** Department of Pharmacology, Faculty of Pharmaceutical Sciences, Toho University, 2-2-1 Miyama Funabashi, Chiba 274-8510, Japan; 3022005s@st.toho-u.jp (T.S.); mahiru-s@iuhw.ac.jp (M.S.); obaaiko2@gmail.com (A.O.); shogo.hamaguchi@phar.toho-u.ac.jp (S.H.); htanaka@phar.toho-u.ac.jp (H.T.)

**Keywords:** pulmonary vein cardiomyocytes, automaticity, intracellular Na^+^, Ca^2+^ overload, late I_Na_, Na^+^/Ca^2+^ exchanger

## Abstract

The effects of enhanced late I_Na_, a persistent component of the Na^+^ channel current, on the intracellular ion dynamics and the automaticity of the pulmonary vein cardiomyocytes were studied with fluorescent microscopy. Anemonia viridis toxin II (ATX- II), an enhancer of late I_Na_, caused increases in the basal Na^+^ and Ca^2+^ concentrations, increases in the number of Ca^2+^ sparks and Ca^2+^ waves, and the generation of repetitive Ca^2+^ transients. These phenomena were inhibited by eleclazine, a blocker of the late I_Na_; SEA0400, an inhibitor of the Na^+^/Ca^2+^ exchanger (NCX); H89, a protein kinase A (PKA) inhibitor; and KN-93, a Ca^2+^/calmodulin-dependent protein kinase II (CaMKII) inhibitor. These results suggest that enhancement of late I_Na_ in the pulmonary vein cardiomyocytes causes disturbance of the intracellular ion environment through activation of the NCX and Ca^2+^-dependent enzymes. Such mechanisms are probably involved in the ectopic electrical activity of the pulmonary vein myocardium.

## 1. Introduction

The present study was undertaken to clarify the intracellular mechanisms for the induction of automaticity in the pulmonary vein myocardium by the persistent component of the voltage-dependent Na^+^ channel current. The pulmonary vein is the blood vessel for the blood flow from the lung to the heart. Spontaneous pulsation of the pulmonary vein independent of the cardiac pacemaker has been repeatedly observed in various animal species [1,2,3]. This is due to a myocardial layer in the pulmonary vein wall connected to the left atrial myocardium capable of generating spontaneous or triggered action potentials. Since the clinical report that the automatic activity of the pulmonary vein myocardium plays a central role in the generation and maintenance of atrial fibrillation [4,5], the automaticity of the pulmonary vein myocardium has received attention as a therapeutic target and has been studied in various animal species [1,2,6,7]. The cardiomyocytes of the pulmonary vein myocardium have a smaller inward-rectifying K^+^ channel current density and less repolarizing power than the atrial myocardium [8,9,10], which allows otherwise masked depolarizing currents to induce a gradual depolarization of the membrane. The depolarizing membrane currents contributing to the diastolic depolarization of the pulmonary vein myocardium include the Na^+^/Ca^2+^ exchanger current [11,12], hyperpolarization-activated current [7,10], and the voltage-dependent Na^+^ channel current [13,14], which is the main subject of the present study.

The voltage-dependent Na^+^ channel current (I_Na_) is composed of two components. The transient component, which is referred to as peak I_Na_, is activated by rapid depolarizations from the resting membrane potential range and rapidly inactivates within a few milliseconds. Peak I_Na_ has a large current density and is responsible for the rapid upstroke and conduction of the action potential in the working myocardium [15]. After the inactivation of the peak I_Na_, a small fraction of the I_Na_ flows persistently [16]; such current is referred to as late I_Na_, persistent I_Na,_ or sustained I_Na_ [17]. Such a persistent Na^+^ current is also observed during a slow depolarization in the negative voltage range and has been suggested to contribute to abnormal automaticity underlying cardiac arrhythmia including atrial fibrillation [18,19]. Although the amplitude of late I_Na_ is small in the normal myocardium, it may be enhanced under various acquired and congenital conditions related to myocardial automaticity such as heart failure, ischemia, and arrhythmia including atrial fibrillation [20,21,22]. In the pulmonary vein myocardium, whose resting or maximum diastolic potential is about −70 mV, less negative than the −80 to −90 mV of the working myocardium [8,9], the late I_Na_ can flow depending on the situation. It was previously reported in the pulmonary vein myocardium that anemonia viridis toxin II (ATX-II), an enhancer of late I_Na_, increased the pacemaker depolarization slope, as well as the automatic firing frequency [13,23]. Agents with inhibitory effects on late I_Na_, such as GS-458967, reduced the pacemaker depolarization slope and automatic activity in the pulmonary vein myocardium [13]. Thus, blockade of late I_Na_ appears to be an effective pharmacological strategy for the treatment of atrial fibrillation.

While late I_Na_ can directly contribute to depolarization as a depolarizing current, it may also cause disturbances in intracellular ion homeostasis and consequently lead to arrhythmic activity. An increase in intracellular Na^+^ concentration through inhibition of the Na^+^/K^+^-ATPase by ouabain induced automatic electrical activity in the pulmonary vein myocardium, which was accompanied by an increase in the number of Ca^2+^ sparks and Ca^2+^ waves; these were both inhibited by pretreatment with a selective NCX inhibitor, SEA0400 [11]. Thus, a probable explanation for the induction of electrical activity by an increase in intracellular Na^+^ concentration in the pulmonary vein myocardium is the induction of Ca^2+^ overload through activation of Ca^2+^ influx through NCX [11]. Ca^2+^ overload is reported to cause hyperactivation of Ca^2+^-dependent enzymes such as Ca^2+^/calmodulin -dependent protein kinase II (CaMKII) and protein kinase A (PKA), and leads to arrhythmias in ventricular muscle [17,24,25,26]. If an enhancement of the late I_Na_ can induce automatic activity in the pulmonary vein myocardium through similar mechanisms, their inhibitors would be promising as drugs for the treatment of atrial fibrillation caused by pulmonary vein automaticity.

In the present study, fluorescent ion measurements and pharmacological studies were conducted with isolated pulmonary vein cardiomyocytes to clarify the following points. Firstly, does enhancement of the late I_Na_ in isolated pulmonary vein myocytes induce automatic electrical activity? Secondly, does the enhancement of the late I_Na_ cause intracellular Ca^2+^ overload through NCX function? Finally, are Ca^2+^-dependent enzymes involved in the induction of automatic activity? Given the positive results obtained, their implication for drug selection and development was discussed.

## 2. Results

### 2.1. The Spontaneous and Evoked Ca^2+^ Transients of Isolated Pulmonary Vein Cardiomyocytes

**Figure 1:**
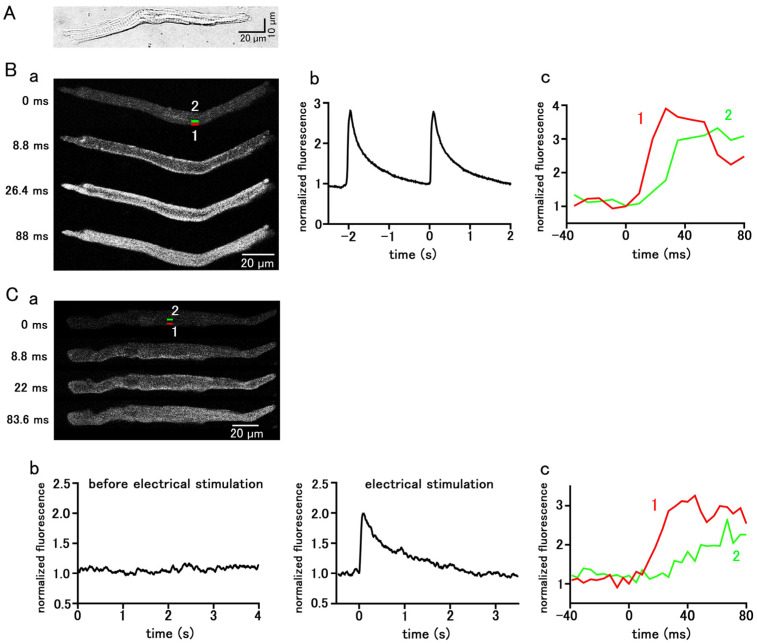
Spontaneous and stimulation-evoked Ca^2+^ transients in pulmonary vein myocytes. (**A**) A typical differential interference contrast (DIC) image. (**B**,**C**) Spontaneous (**B**) and electrical-stimulation-evoked (**C**) Ca^2+^ transients. Typical x−y images of a cell loaded with Fluo−4/AM (**B**(**a**),**C**(**a**)). Time course of fluorescence intensity of the whole cell (**B**(**b**),**C**(**b**)) and quantified in rectangular (1 × 4 µm) regions located at 0–1 µm (1 red) and 4–5 µm (2 green) from the sarcolemma (**B**(**c**),**C**(**c**)), as shown in the top panel in (**B**(**a**),**C**(**a**)).

Pulmonary vein cardiomyocytes isolated from guinea pigs were spindle-shaped and had transverse striations. (Figure 1A). They were loaded with the Ca^2+^-sensitive fluorescent probe, Fluo-4, and observed with a confocal microscope. Among the 787 isolated guinea pig pulmonary vein cardiomyocytes examined, 220 cells showed spontaneous Ca^2+^ transients, which is a rapid rise in the Ca^2+^ concentration in the entire subsarcolemmal cytoplasm (Figure 1B); the average firing frequency of the Ca^2+^ transient was 1.85 ± 0.13 times in 5 s. During the early phase of the Ca^2+^ transient, fluorescence intensity increased from the subsarcolemmal region and then spread to the cell interior (Figure 1B). In the quiescent pulmonary vein cardiomyocytes, action potentials were elicited by field electrical stimulation, and the resulting Ca^2+^ transients were observed; the rise in Ca^2+^ concentration started from the subsarcolemmal region and spread to the central region of the cell (Figure 1C).

### 2.2. Effect of Enhanced Late I_Na_ on Automatic Activity and Intracellular Ca^2+^ Dynamics

**Figure 2:**
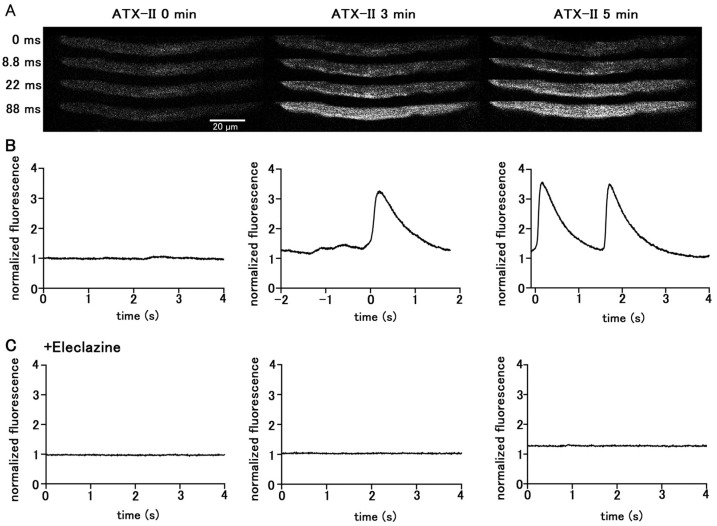
Induction of Ca^2+^ transients by ATX-II in quiescent pulmonary vein cardiomyocytes. Typical x−y images (**A**) and time course (**B**) of a cell loaded with Fluo−4 before ATX-II treatment and at 3 and 5 min after treatment with 100 nM ATX-II. Pretreatment of eleclazine (10 µM) inhibited the induction of automatic activity (**C**).

Treatment of quiescent pulmonary vein myocytes with 100 nM ATX-II induced automatic Ca^2+^ transients. Among the 40 isolated pulmonary vein cardiomyocytes examined, automatic Ca^2+^ transients were induced in 18 cells. The firing frequency of Ca^2+^ transients increased in a time-dependent manner (Figure 2); the average frequency was 1.25 ± 0.25 times in 5 s at 3 min and 2.25 ± 0.25 times in 5 s at 5 min. Regarding the automatic Ca^2+^ transient induced by ATX-II, the rise in the Ca^2+^ concentration first occurred at the subsarcolemmal region and spread to the central region of the cell, similarly to electrical-stimulation-evoked Ca^2+^ transients. Inhibition of late I_Na_ by pretreatment with 10 µM eleclazine significantly reduced the incidence of Ca^2+^ transients induced by ATX-II (Figure 2). Such induction of automatic activity by ATX-II was less frequently observed in atrial cardiomyocytes. Among the 56 isolated atrial cardiomyocytes examined, automatic Ca^2+^ transients were induced only in seven cells.

**Figure 3:**
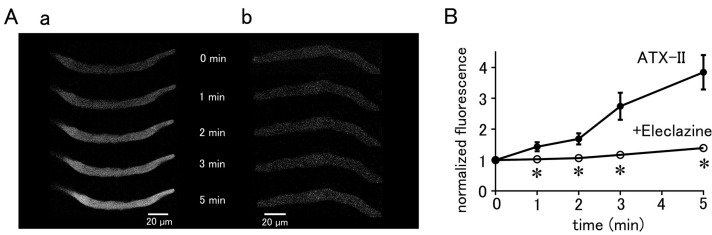
ATX-II-induced changes in intracellular Ca^2+^ concentration. (**A**) Typical x−y images of pulmonary vein cardiomyocytes loaded with Fluo−4 after treatment with 100 nM ATX-II alone (**a**) and in the presence of 10 μM eleclazine (**b**). (**B**) Time course of the changes in basal Ca^2+^ concentration induced by 100 nM ATX-II alone (closed circles) and in the presence of 10 μM eleclazine (open circles). Each point with vertical bars represents the mean ± S.E.M of 30–40 cells. Asterisks indicate significant differences (*p* < 0.05) from the corresponding values in ATX-II-treated cells.

The basal Ca^2+^ fluorescence intensity of the cells was increased by ATX-II in a time-dependent manner, regardless of the induction of automatic Ca^2+^ transients (Figure 3). Pretreatment of the cells with 10 µM eleclazine for 5 min significantly suppressed the ATX-II-induced increase in basal Ca^2+^ concentration (Figure 3).

### 2.3. Effect of Enhanced Late I_Na_ on Intracellular Na^+^ Concentration

**Figure 4:**
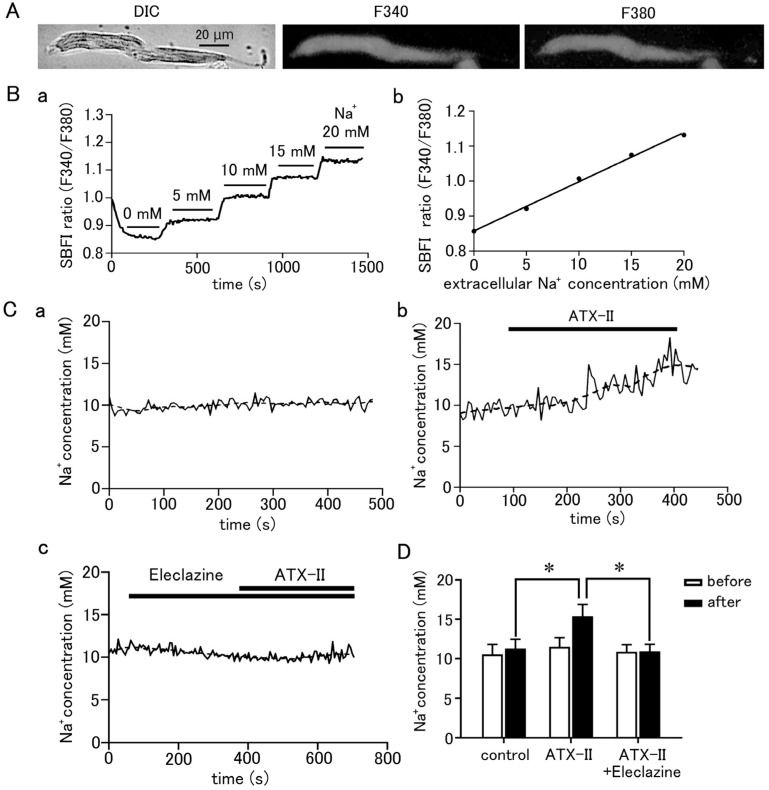
Quantitative measurement of changes in intracellular Na^+^ concentration in pulmonary vein myocardium induced by ATX-II. (**A**) Differential interference contrast (DIC) images and fluorescence images excited at 340 nm and 380 nm in pulmonary vein cardiomyocytes loaded with SBFI. (**B**) Determination of intracellular Na^+^ concentration in pulmonary vein cardiomyocytes. After measuring the Na^+^ fluorescence ratio, calibration of the fluorescence ratio to Na^+^ concentration was performed as described in the methods section (**B**(**a**)); the calibration curve was prepared by plotting the fluorescence intensity ratio of SBFI obtained in (**B**(**a**)) for each extracellular Na^+^ concentration (**B**(**b**)). (**C**) Effect of 100 nM ATX-II on intracellular Na^+^ concentration. Time course of Na^+^ concentration for time control (**C**(**a**)), ATX-II (**C**(**b**)), and ATX-II in the presence of 10 μM eleclazine (**C**(**c**)). The solid line and the dashed line represent the Na^+^ concentration obtained every 5 s and its 15-point moving average, respectively. (**D**) Intracellular Na^+^ concentration at 5 min after ATX-II treatment. Each column with vertical bars represents the mean ± S.E.M. of 7 cells. Asterisks indicate significant differences (*p* < 0.05) from the corresponding values.

To measure the intracellular Na^+^ concentration, the pulmonary vein cardiomyocytes were loaded with SBFI/AM and observed by epifluorescence microscopy. SBFI was excited at 340 nm when bound to Na^+^ and at 380 nm when unbound, and the ratio of their fluorescence (F_340_/F_380_) can be taken to quantitatively measure Na^+^ concentration. Fluorescence excited at either wavelength was found to be homogeneously distributed in the cell (Figure 4A). The intracellular Na^+^ concentration could be quantified using a calibration curve based on a staircase graph of F_340_/F_380_ obtained by adjusting the Na^+^ concentrations in the extracellular fluid to 0, 5, 10, 15, and 20 mM (Figure 4B). The intracellular Na^+^ concentration in quiescent pulmonary vein cardiomyocytes was 11.19 ± 0.16 mM (*n* = 23). Intracellular Na^+^ concentration was increased by 3.91 ± 0.7 mM in 5 min by treatment with 100 nM ATX-II (*n* = 7; Figure 4C,D). This was significantly higher than the 0.73 ± 0.1 mM increase in Na^+^ concentration seen in the time control (*n* = 7). Pretreatment with 10 µM eleclazine significantly suppressed the ATX-II-induced increase in Na^+^ concentration; the ATX-II-induced increase in Na^+^ concentration was 0.06 ± 0.2 mM (*n* = 7).

### 2.4. Contribution of NCX to Automatic Activity

**Figure 5:**
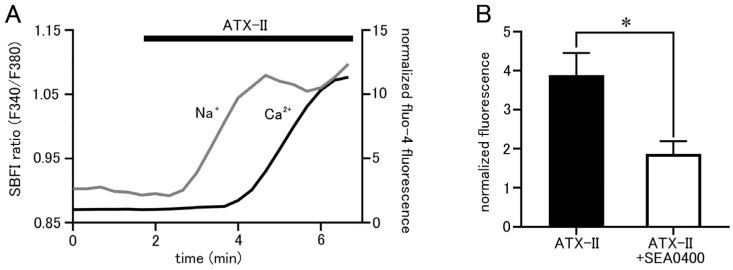
ATX-II-induced changes in intracellular ion concentrations and the involvement of NCX. (**A**) Time course of respective fluorescence intensity in cells loaded with SBFI and Fluo−4. The gray line shows the fluorescence intensity ratio of SBFI, and the black line shows the fluorescence intensity of Fluo-4. Note that ATX-II (100 nM) induced an increase in the fluorescence intensity ratio of SBFI, followed by an increase in Fluo-4 fluorescence intensity. (**B**) Effect of SEA0400 (1 μM) on the elevation of basal Ca^2+^ concentration induced by ATX-II. Each column with vertical bars represents the mean ± S.E.M. of 30–40 cells. Asterisks indicate significant differences (*p* < 0.05) from the corresponding values in ATX-II-treated cells.

Isolated pulmonary vein cardiomyocytes were loaded with SBFI and Fluo-4, and intracellular Na^+^ and Ca^2+^ concentrations were simultaneously measured by epifluorescence microscopy. ATX-II (100 nM) caused increases in both Na^+^ and Ca^2+^ concentrations, but it took 1.23 ± 0.2 min for the Na^+^ concentration to reach half of its peak value and 3.46 ± 0.2 min for the Ca^2+^ concentration (*n* = 5; Figure 5A). Thus, ATX-II induced increases in intracellular Na^+^ and Ca^2+^ concentrations sequentially in this order. Furthermore, pretreatment with 1 µM of SEA0400, a selective NCX inhibitor, significantly reduced the ATX-II-induced increase in basal Ca^2+^ concentration (Figure 5B).

### 2.5. ATX-II-Induced Ca^2+^ Oscillation and Involvement of Ca^2+^-Dependent Enzymes

**Figure 6:**
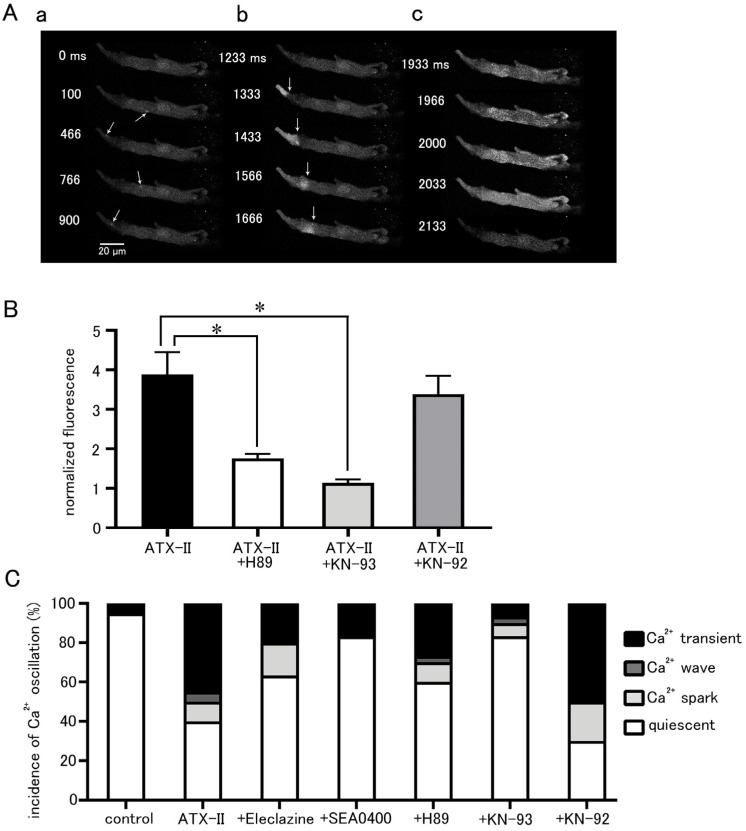
Induction of intracellular Ca^2+^ oscillation by ATX-II and the effects of pharmacological agents. (**A**) Typical x−y images of a Fluo-4-loaded pulmonary vein cardiomyocyte after ATX-II treatment (100 nM) showing Ca^2+^ sparks (arrows; (**a**)), a Ca^2+^ wave (arrows indicate wavefront of Ca^2+^ wave; (**b**)), and a Ca^2+^ transient (**c**). The images in ((**a**–**c**)) were obtained in this order in the same cell. (**B**) The effects of H89 (5 μM), KN-93 (3 μM), and KN-92 (3 μM) on the ATX-II-induced increase in basal Ca^2+^ concentration. Asterisks indicate significant differences (*p* < 0.05) from the corresponding control values. (**C**) The effects of eleclazine (10 μM), H89 (5 μM), KN-93 (3 μM), and KN-92 (3 μM) on the incidence of ATX-II-induced Ca^2+^ oscillations. Each column with vertical bars in (**B**,**C**) represents the mean ± S.E.M of 20–40 cells.

The effect of ATX-II on intracellular Ca^2+^ dynamics appeared in the form of an increase in basal Ca^2+^ concentration, Ca^2+^ sparks, and Ca^2+^ waves as well as Ca^2+^ transients (Figure 6A). These Ca^2+^ oscillations often occurred after 3 min of ATX-II treatment. The increase in basal Ca^2+^ concentration by 100 nM ATX-II occurred in all cells examined; it was suppressed by pretreatment with 5 µM H89 (*n* = 30), a PKA inhibitor, and 3 µM KN-93 (*n* = 30), a CaMKII inhibitor (Figure 6B). Such suppression was not observed with 3 µM KN-92 (*n* = 20), an inert analog of KN-93. The percentage of cells in which these Ca^2+^ oscillations were induced by ATX-II was significantly higher than the time control, and Ca^2+^ transients were observed in approximately half of the cells (Figure 6C). Pretreatment with eleclazine, SEA0400, H89, and KN-93 reduced the incidence of Ca^2+^ oscillations induced by ATX-II (Figure 6C).

## 3. Discussion

### 3.1. ATX-II-Induced Changes in Intracellular Ca^2+^ Concentration

The present study was undertaken to examine whether a pharmacological enhancement of the late I_Na_ can induce automatic activity in the pulmonary vein cardiomyocytes and to clarify the intracellular mechanisms involved. We first confirmed the relationship between action potential generation and the Ca^2+^ transient, a temporal increase in Ca^2+^ concentration throughout the cell. Both in the spontaneous Ca^2+^ transients and action-potential evoked Ca^2+^ transients, the rise in Ca^2+^ concentration occurred initially in the subsarcolemmal region and then spread to the cell center (Figure 1). This is a characteristic feature of Ca^2+^ transients in cardiomyocytes lacking T-tubules, including the atrial and pulmonary vein cardiomyocytes [27]. In cells lacking T-tubules, the trans-sarcolemmal Ca^2+^ influx elicited by an action potential first triggers Ca^2+^ release from the Ca^2+^ release channel (ryanodine receptor) in the sarcoplasmic reticulum (SR) in the subsarcolemmal region [27,28,29]. Next, in the central region lacking T-tubules, a propagated Ca^2+^-induced Ca^2+^ release mechanism in the SR activates the Ca^2+^ release from SR [28,29]. This pattern of rise in Ca^2+^ indicates the firing of action potentials. The same pattern was observed in ATX-II-induced Ca^2+^ transients (Figure 2), indicating that they were accompanied by action potentials. A characteristic feature of ATX-II-induced Ca^2+^ transients is that they were preceded by a gradual rise in basal Ca^2+^ concentration (Figure 3). This suggests that the rise in Ca^2+^ concentration is a cause of action potential generation. The rise in basal Ca^2+^ concentration, as well as the Ca^2+^ transient, was inhibited by eleclazine, confirming that both phenomena were caused by enhancement of late I_Na_ (Figure 2 and Figure 3).

### 3.2. NCX Is Involved in ATX-II-Induced Increases in Intracellular Na^+^ and Ca^2+^ Concentrations

ATX-II caused an increase in intracellular Na^+^ concentration in quiescent pulmonary vein cardiomyocytes, which was inhibited by eleclazine (Figure 4). These results also indicated that late I_Na_ could be activated at negative membrane potentials near the resting potential. This is consistent with our previous results with voltage-clamped pulmonary vein cardiomyocytes that the late I_Na_ can flow at membrane potentials near the resting potential of about −70 mV [13,30]. On the other hand, in the ventricular cardiomyocytes, ATX-II increased the Na^+^ concentration in electrically stimulated cardiomyocytes but had no effects on Na^+^ concentration in quiescent cardiomyocytes [31]. Our results also showed that ATX-II tended not to induce automatic activity in atrial cardiomyocytes. This is probably because the resting membrane potential of the atrial and ventricular myocardium of about −80 to −90 mV is more negative than that of the pulmonary vein myocardium [8,9]; the late I_Na_ is unlikely to flow near the resting membrane potential in the working myocardium. The pulmonary vein myocardium has a smaller inward rectifying K^+^ channel current density and less repolarizing power than the working myocardium [8,9,10]; this allows the membrane potential to stay in a less negative voltage range in which the late I_Na_ can flow.

Simultaneous measurement of intracellular Na^+^ and Ca^2+^ concentrations revealed that the ATX-II-induced increase in intracellular Na^+^ concentration was followed by an increase in Ca^2+^ concentration (Figure 5A); this order suggested the involvement of the reverse-mode NCX. The NCX has two modes of action: the forward mode, in which Na^+^ is taken into the cell and Ca^2+^ is excreted, and the reverse mode, in which Na^+^ is excreted from the cell and Ca^2+^ is taken into the cell. The forward mode is dominant in normal cardiomyocytes, but under conditions with elevated intracellular Na^+^ concentration such as ischemia–reperfusion or cardiac glycoside treatment, the NCX can function in the reverse mode and cause an increase in intracellular Ca^2+^ concentration [32]. In the present study, the treatment of quiescent cardiomyocytes with ATX-II increased the Na^+^ concentration from 11 to 15 mM, and, as mentioned above, the resting membrane potential of the pulmonary vein myocardium is less negative than that of the working myocardium [8,9]; these are conditions which favor Ca^2+^ entry through the reverse-mode NCX. Some reports suggested that the reverse-mode NCX may be more dominant at intracellular Na^+^ concentrations above 12 mM [33,34]. The result that SEA0400, a selective inhibitor of NCX, inhibited the increase in basal Ca^2+^ concentration by ATX-II (Figure 5B) also confirmed the involvement of NCX. Among the three known types of NCX, the expression of the SEA0400-sensitive NCX1 was reported in the pulmonary vein myocardium of rats [12].

### 3.3. ATX-II Ca^2+^ Overload Triggers Further Spontaneous Activity

ATX-II induced not only an increase in basal Ca^2+^ concentration but also Ca^2+^ oscillations such as Ca^2+^ sparks and Ca^2+^ waves, which are local non-propagating increases in Ca^2+^ concentration that occur independently of action potentials [28,29]. Ca^2+^ sparks, which are the unit of Ca^2+^ release from the SR Ca^2+^ release channel, occur in a small area of 1 to 2 μm in diameter, while on Ca^2+^ waves, the Ca^2+^ release propagates within the cell at a velocity of about 100 μm/s [27]. Confocal imaging in pulmonary vein tissue showed that Ca^2+^ waves, as well as Ca^2+^ sparks, do not propagate between connected cells, which confirms that they occur independently of action potential firing [35]. Ca^2+^ waves and Ca^2+^ sparks are considered to reflect spontaneous Ca^2+^ leaks from the sarcoplasmic reticulum which are enhanced in Ca^2+^ overload myocytes [36,37,38]. The increase in cytoplasmic Ca^2+^ concentration not only stimulates the SR Ca^2+^ release channel from the cytoplasmic side through the Ca^2+^-induced Ca^2+^ release mechanism but also causes an increase in Ca^2+^ concentration in the lumen of the SR, which affects the Ca^2+^ release channel and increases the Ca^2+^ sparks and Ca^2+^ waves. The abnormal increase in cytoplasmic Ca^2+^ causes hyperactivation of voltage-dependent ion channels, Ca^2+^-activated channels, and Ca^2+^-driven transporters, which leads to spontaneous electrical activity and arrhythmias [39]. The induction of Ca^2+^ sparks, Ca^2+^ waves, and Ca^2+^ transients in the pulmonary vein myocardium was also observed with ouabain, which increases intracellular Na^+^ concentration through inhibition of the Na^+^/K^+^ ATPase [11]. We also observed that the Ca^2+^ waves and Ca^2+^ transients were suppressed by carbachol, which hyperpolarized the resting membrane potential of the pulmonary vein myocardium to the same level as the working myocardium [8]. Thus, less repolarizing power of the pulmonary vein cardiomyocytes appears to play a permissive role in the generation of Ca^2+^ overload and the subsequent induction of automatic activity. 

**Figure 7:**
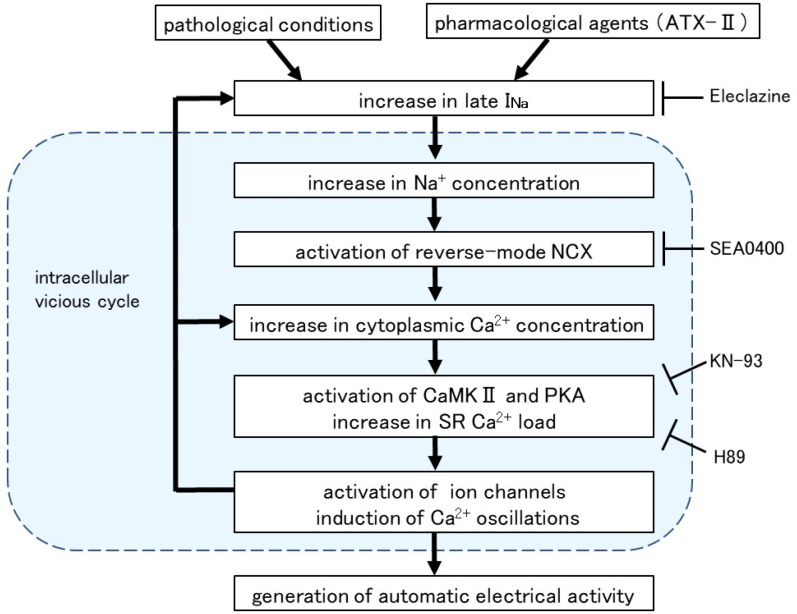
Intracellular mechanism for the induction of automatic activity by enhanced late I_Na_. Arrows denote stimulation and T-shaped lines indicate inhibition.

Intracellular Ca^2+^ overload in cardiomyocytes not only causes alterations in ion channels and transporters but also causes activation of various Ca^2+^-dependent enzymes [40]. The ATX-II-induced increase in basal Ca^2+^ concentration and generation of Ca^2+^ oscillation was suppressed in the presence of H89, a PKA inhibitor, and KN-93, a CaMKII inhibitor (Figure 6). A similar suppression of automatic activity after inhibition of CaMKII and PKA has been reported in rabbit pulmonary vein myocardium [41]. The activation of adenylate cyclase (AC) and PKA was reported in cardiomyocytes from the rat pulmonary vein [42] and guinea pig atria [43]. CaMKII, a multifunctional serine/threonine protein kinase is activated by the Ca^2+^/calmodulin complex when intracellular Ca^2+^ levels are increased [44,45]. CaMKII could phosphorylate the Na^+^ channels and Ca^2+^ channels [46], which could cause membrane depolarization. The hyperphosphorylation of the respective target sites on ryanodine receptors and phospholamban by CaMKII and PKA triggered a significant increase in Ca^2+^ leaks from SR [43]. Thus, enhancement of late I_Na_ and the resulting intracellular Ca^2+^ overload causes hyperactivation of various Ca^2+^-dependent ion channels, transporters, and enzymes, which in turn causes further accumulation of intracellular Ca^2+^ (Figure 7).

### 3.4. Clinical Implications of This Study

The present study showed that induction of pulmonary vein automaticity by enhancement of the late I_Na_ is partly mediated by mechanisms including the NCX and enzymes such as CaMKII and PKA. This implies that inhibitors of these mechanisms may be promising as drugs for the treatment of atrial fibrillation caused by pulmonary vein automaticity. Ranolazine, which selectively blocks the late I_Na_, was shown to be anti-arrhythmic in human patients in some situations such as post-operative atrial fibrillation [47]; the mechanisms of action still remain to be clarified because the drug also has effects on many other ion channels and the α-adrenoceptor. Agents with higher selectivity towards late I_Na_ such as GS-458967 [13] and NCC-3902 [30] have been shown to inhibit pulmonary vein automaticity and to be effective against animal models of atrial fibrillation but have not been applied to human patients at present. Some of the class I antiarrhythmic drugs were shown to have inhibitory effects on the late I_Na_ and reduce the diastolic depolarization slope of the isolated guinea pig pulmonary vein myocardium [48]; whether such effects contribute to the antiarrhythmic effect of these drugs in human patients is unknown at present. A novel inhibitor of the NCX, SAR296968, was reported to reduce the frequency of Ca^2+^ sparks in isolated human atrial cardiomyocytes [49]; whether such effects can lead to antiarrhythmic effects in vivo awaits further investigation because the effect of NCX inhibitors appears to largely vary depending on the experimental condition [50,51]. Some of the class I antiarrhythmic drugs were reported to have inhibitory effects on the NCX, but only at concentrations higher than the therapeutic blood concentration [52]. A CaMKII inhibitor, AS105, which is more potent and selective than KN-93, was reported to inhibit SR Ca^2+^ leaks in human atrial cardiomyocytes [53,54]. Whether the mechanisms clarified in this study are involved in the pulmonary vein automaticity of human patients and whether these inhibitors are effective in clinical practice await further investigation.

### 3.5. Conclusions

The present results suggested that enhancement of late I_Na_ in pulmonary vein cardiomyocytes causes Ca^2+^ overload through the reverse-mode NCX and induces automatic activity. Such mechanisms are particularly likely to occur in the pulmonary vein myocardium, whose repolarizing membrane current density is lower than the working myocardium.

## 4. Materials and Methods

### 4.1. General

All experiments were performed in compliance with the Guiding Principles for the Care and Use of Laboratory Animals of the Japanese Pharmacological Society and were approved by the Toho University Animal Care and User Committee (21-41-507, 7 May 2022). 

### 4.2. Isolation of Pulmonary Vein Cardiomyocytes

Pulmonary vein cardiomyocytes were obtained according to the methods described in our previous study [13,27,30]. Male Hartley guinea pigs (weight, 300–450 g) were anesthetized with isoflurane, and the hearts with lungs were isolated and perfused via the aorta with a Tyrode’s solution of the following composition (mM concentration): NaCl 143, KCl 4, MgCl_2_ 0.5, CaCl_2_ 1.8, NaH_2_PO_4_ 0.33, glucose 5.5, and HEPES 5 (pH 7.4, gassed with 100% O_2_, and warmed to 36 °C). The heart was further perfused successively with nominally Ca^2+^-free Tyrode’s solution and the same solution containing 0.1 mg/mL protease (type XIV; Sigma-Aldrich, St. Louis, MO, USA) and 0.5 mg/mL collagenase (YK-102; Yakult, Tokyo, Japan) for about 20 min. After the washout of the enzymes, the cardiomyocytes were isolated by gentle disruption of the pulmonary vein.

### 4.3. Measurement of Intracellular Ca^2+^ Dynamics

For the measurement of intracellular Ca^2+^ dynamics, the isolated pulmonary vein cardiomyocytes were loaded with 5 µM Fluo-4/AM (Dojindo, Kumamoto, Japan) for about 30 min and superfused with the Tyrode’s solution described above at room temperature. The cardiomyocytes were observed with a rapid-scanning laser confocal microscope (A1R; Nikon Corporation, Tokyo, Japan) so that the Ca^2+^ sparks and Ca^2+^ waves could be detected. The objective lens was Apochromat ×40, 1.15 numerical aperture (water immersion). The excitation wavelength was 488 nm, and the emission in the wavelength range of 500 to 550 nm was detected. The data were analyzed with computer software, NIS elements (Nikon Corporation). The fluorescence intensity of Fluo-4 at each time point was normalized against the basal intensity. The scanning was performed every 4.4 ms at 1024 × 64 pixels or every 8.8 ms at 1024 × 128 pixels. For the triggering of action potentials, field electrical stimulation by a rectangle voltage pulse of 3 ms duration was applied to cells through a platinum wire electrode paired with an electric stimulator (SEN-3303; Nihon Kohden Corporation, Tokyo, Japan).

### 4.4. Measurement of Intracellular Na^+^ Concentration

Isolated cardiomyocytes were loaded with SBFI (5 μM SBFI/AM) and 0.05% pluronic F-127 at 36 °C (Invitrogen, Carlsbad, CA, USA), and the total Na^+^ fluorescence from single cardiomyocytes was measured with epifluorescence microscopy. The cells were excited at 340 and 380 nm from a xenon lamp, and the emission (>500 nm) was separated with a dichroic mirror, detected by a cooled CCD camera (C6790, Hamamatsu Photonics, Shizuoka, Japan) at a time resolution of 5 s, and ratioed after correction of background fluorescence (Aquacosmos software, version 2.52, Hamamatsu Photonics, Shizuoka, Japan). A cooled CCD camera was used because a rapid-scanning laser confocal microscope is not suitable for excitation using two wavelengths. In situ calibration of SBFI was performed by exposing the myocytes to various extracellular Na^+^ solutions in the presence of 10 μM ouabain, 2 µM gramicidin, and 40 μM monensin. The Na^+^ concentration was increased from 0 mM to 20 mM in 5 mM steps.

### 4.5. Simultaneous Measurement of Intracellular Ca^2+^ and Na^+^ Concentrations

For the simultaneous measurement of intracellular Ca^2+^ and Na^+^ concentration, the isolated pulmonary vein cardiomyocytes were loaded with Fluo-4/AM and SBFI/AM (see above). The cells were excited at 480 nm for Fluo-4 from an LED lamp (COLIBRI, Carl Zeiss, Oberkochen, Germany), and 340 and 380 nm for SBFI from a mercury lamp (HXP120V, Zeiss, Oberkochen, Germany), and the emission (>500 nm) was separated with a dichroic mirror, detected by a digital camera (AxioCam MRm, Carl Zeiss, Oberkochen, Germany) at a time resolution of 10 s, and ratioed after correction of background fluorescence (Zen Pro, Carl Zeiss).

### 4.6. Chemicals

ATX-II and H89 were purchased from Alomone Labs (Jerusalem, Israel); gramicidin, monensin, ouabain, and SEA0400 were purchased from Sigma-Aldrich (St. Louis, USA); eleclazine was purchased from Cosmo Bio (Tokyo, Japan); and KN-93 and KN-92 were purchased from Wako Pure Chemical Industries Ltd. (Osaka, Japan). ATX-II was dissolved in distilled water, and monensin was dissolved in ethanol. All other chemicals were dissolved in dimethyl sulfoxide.

### 4.7. Data Analytics and Statistics

All data were expressed as the mean ± standard error of the mean (S.E.M.). Statistical significance between means was evaluated by Welch’s *t*-test, or one-way repeated-measures ANOVA followed by Tukey’s or Dunnett’s multiple comparisons. A *p-value* less than 0.05 was considered statistically significant.

## Data Availability

Data are contained within the article.
